# The Dominant Australian Community-Acquired Methicillin-Resistant *Staphylococcus aureus* Clone ST93-IV [2B] Is Highly Virulent and Genetically Distinct

**DOI:** 10.1371/journal.pone.0025887

**Published:** 2011-10-03

**Authors:** Kyra Y. L. Chua, Torsten Seemann, Paul F. Harrison, Shaun Monagle, Tony M. Korman, Paul D. R. Johnson, Geoffrey W. Coombs, Brian O. Howden, John K. Davies, Benjamin P. Howden, Timothy P. Stinear

**Affiliations:** 1 Department of Microbiology, Monash University, Clayton, Australia; 2 Department of Microbiology and Immunology, University of Melbourne, Parkville, Australia; 3 Victorian Bioinformatics Consortium, Monash University, Clayton, Australia; 4 Department of Infectious Diseases, Austin Centre for Infection Research, Austin Health, Heidelberg, Australia; 5 Department of Microbiology, Austin Health, Heidelberg, Australia; 6 Department of Anatomical Pathology, Eastern Health, Box Hill, Australia; 7 Department of Infectious Diseases, Monash Medical Centre, Clayton, Australia; 8 Department of Microbiology and Infectious Diseases, PathWest Laboratory Medicine WA, Royal Perth Hospital, Perth, Australia; 9 Microsurgical Consultants, Blackburn, Australia; University of Liverpool, United Kingdom

## Abstract

Community-associated methicillin-resistant *Staphylococcus aureus* (CA-MRSA) USA300 has spread rapidly across North America, and CA-MRSA is also increasing in Australia. However, the dominant Australian CA-MRSA strain, ST93-IV [2B] appears distantly related to USA300 despite strikingly similar clinical and epidemiological profiles. Here, we compared the virulence of a recent Australian ST93 isolate (JKD6159) to other MRSA, including USA300, and found that JKD6159 was the most virulent in a mouse skin infection model. We fully sequenced the genome of JKD6159 and confirmed that JKD6159 is a distinct clone with 7616 single nucleotide polymorphisms (SNPs) distinguishing this strain from all other *S. aureus* genomes. Despite its high virulence there were surprisingly few virulence determinants. However, genes encoding α-hemolysin, Panton-Valentine leukocidin (PVL) and α-type phenol soluble modulins were present. Genome comparisons revealed 32 additional CDS in JKD6159 but none appeared to encode new virulence factors, suggesting that this clone's enhanced pathogenicity could lie within subtler genome changes, such as SNPs within regulatory genes. To investigate the role of accessory genome elements in CA-MRSA epidemiology, we next sequenced three additional Australian non-ST93 CA-MRSA strains and compared them with JKD6159, 19 completed *S. aureus* genomes and 59 additional *S. aureus* genomes for which unassembled genome sequence data was publicly available (82 genomes in total). These comparisons showed that despite its distinctive genotype, JKD6159 and other CA-MRSA clones (including USA300) share a conserved repertoire of three notable accessory elements (SSC*mec*IV, PVL prophage, and pMW2). This study demonstrates that the genetically distinct ST93 CA-MRSA from Australia is highly virulent. Our comparisons of geographically and genetically diverse CA-MRSA genomes suggest that apparent convergent evolution in CA-MRSA may be better explained by the rapid dissemination of a highly conserved accessory genome from a common source.

## Introduction

Infections with community-associated methicillin-resistant *Staphylococcus aureus* (CA-MRSA) in patients with no contact with the hospital environment are now well described worldwide [1]. In some regions such as North America, the CA-MRSA phenomenon has become particularly well defined. Here, the epidemic CA-MRSA strain USA300 is the most common cause of skin infections in patients presenting to the emergency department [2]. Much of the work performed thus far to understand the CA-MRSA phenomenon has focused on USA300 and these studies have demonstrated the importance of virulence determinants in both the staphylococcal accessory and core genome such as Panton-Valentine leukocidin (PVL), α-hemolysin (Hla) and α-type phenol soluble modulins (PSMs) [3], [4], [5], [6], [7], [8], [9], [10].

In contrast to healthcare-associated MRSA where a limited number of clones are prevalent, CA-MRSA strains are polyclonal with an epidemiological association between clone type and geographic origin [1], [11]. Furthermore, minor genetic differences between CA-MRSA strains, even to a single nucleotide, may result in significant differences in the observed virulence of a strain [12]. Hence, while the genomic mediators of virulence have been well defined in USA300, these may not be applicable to other CA-MRSA clones. Therefore, a detailed understanding of the virulence phenotype and virulence determinants in other global clones of CA-MRSA has the potential to provide new and important insights into staphylococcal pathogenesis.

In Australia, *S. aureus* ST93-IV [2B] has recently emerged to become the dominant CA-MRSA clone. First described in the early 2000s, it is colloquially known as the “Queensland” clone [13]. However, since this initial description, ST93-IV [2B] has been reported across Australia and is responsible for the increasing prevalence of all CA-MRSA nationwide [14]. ST93 is associated with skin infection and severe invasive infection including necrotizing pneumonia, deep-seated abscesses, and septicemia [14], [15]. This PVL positive clone is a singleton by MLST eBURST analysis and is therefore distinct to other *S. aureus* clones, including previously sequenced strains [16]. While predominantly an Australian strain, it has recently also been reported in the United Kingdom, with many cases having epidemiological links to Australia [16], [17]. Other less frequent clones of CA-MRSA in Australia include PVL negative ST1-IV [2B] (WA-1) and PVL positive ST30-IV [2B] (SouthWest Pacific, SWP) [14]. The dominant health-care associated clone of MRSA in Australia is multi-resistant ST239-III [3A] [14].

To investigate the apparent enhanced clinical virulence of ST93-IV [2B], we examined a recent clinical isolate (JKD6159) that caused an outbreak of both severe and minor staphylococcal infections within a household, representative of the typical spectrum of disease associated with this clone. We compared the virulence of JKD6159 to three other well-characterized and representative Australian MRSA strains and the epidemic North American strain, USA300 and found that JKD6159 was the most virulent clone in two *in vivo* models.

We next sequenced, fully assembled and annotated the genome of *S. aureus* ST93-IV [2B] (JKD6159) and then sequenced three non-ST93 Australian CA-MRSA clones. We employed comparative genomics against these strains, the 19 published, complete *S. aureus* genomes and 59 unassembled, publically available *S. aureus* genomes to try and identify genetic factors that might explain the recent emergence, and increased virulence of ST93-IV [2B].

## Materials and Methods

### Bacterial strains and culture

The bacterial strains used in this study are described in Table 1. For all experiments, bacteria were grown in brain heart infusion broth (BHI, Oxoid). For growth kinetic experiments, overnight cultures of *S. aureus* were diluted 1∶100 and incubated at 37°C with shaking (180 rpm). Optical density at 600 nm (OD_600_) and viable counts were performed hourly until stationary phase was achieved and at 24 hours after commencement of the experiment. These experiments were performed in triplicate. For the *Galleria mellonella* killing assay and the mouse skin infection assay, *S. aureus* were harvested at the stationary phase of growth after 18 hours incubation (OD_600_ approx. 2.0), washed, diluted and resuspended in PBS. The bacterial inoculum (CFU) and viable counts were determined by plating onto BHI agar and colony enumeration.

**Table 1:** Bacterial strains used in this study.

*S. aureus* strain	Type	Date of Isolate Recovery	Place of Isolate Recovery	Relevant Characteristics	*lukSF-PV*	Reference
JKD6159	ST93-IV [2B]	2004	Melbourne, Australia	Dominant Australian CA-MRSA clone	**+**	This work
JKD6272	ST1-IV [2B]	2002	Melbourne, Australia	Australian CA-MRSA clone	**−**	This work
JKD6260	ST1-IV [2B]	2008	Port Hedland, Australia	Australian CA-MRSA clone	**+**	This work
JKD6177	ST30-IV [2B]	2003	Melbourne, Australia	Australian CA-MRSA clone	**+**	This work
FPR3757 USA300	ST8-IV [2B]	NA	San Francisco, USA	Dominant North American CA-MRSA clone	**+**	[38]
JKD6009	ST239-III [3A]	2002	New Zealand	Dominant Australian hospital-associated MRSA clone, AUS2/3	**−**	[47]

NA: not available.

### DNA Manipulation and Molecular Typing

DNA was extracted using the GenElute kit according to the manufacturer's instructions (Sigma-Aldrich). Detection of *lukSF-PV* was performed as described by Lina *et al.* [18]. MLST was performed as described by Enright *et al.* [19], and Pulsed Field Gel Electrophoresis (PFGE) performed as previously described [20]. SCC*mec* typing was performed as previously described [21]. SCC*mec* nomenclature was used as proposed by the International Working Group on the Classification of Staphylococcal Cassette Chromosome Elements (IWG-SCC) [22]. A Roman numeral indicates the SCC*mec* type with a lowercase letter indicating the subtype. The *ccr* complex and the *mec* complex are indicated by an Arabic numeral and an uppercase letter respectively.

### Wax Moth (*Galleria mellonella)* virulence assay

Final stage instar *G. mellonella* larvae were infected with *S. aureus* and time to death measured as described previously [23]. Approximately 10^6^ CFU of *S. aureus* suspended in 10 µL of PBS was injected into the first left proleg of each larva. Each experimental group consisted of at least 16 larvae. All experiments included biological triplicates. The larvae were incubated at 37°C and assessed daily for six days. Larvae were considered dead when there was no movement in response to stimuli. The control groups were PBS injected and non-injected larvae.

### Mouse skin infection assay

Mice were housed in individually ventilated cages and received food and water ad libitum. Six-week-old female BALB/c mice were anesthetized with intraperitoneal ketamine/xylazine, and inoculated in the right flank by intradermal injection, with 10^8^ CFU of *S. aureus* (in 50 µL of PBS suspension). This dose of *S. aureus* was determined in preliminary experiments as the lower limit of inoculum, which produced consistent dermonecrosis for all strains of *S. aureus* tested. Mice were assessed and weighed daily for five days. On the 5^th^ day, the mice were culled. The infected area of skin and muscle was harvested and these tissues were homogenized and CFU measured by plating onto BHI and colony enumeration. Skin lesion area was quantitated by obtaining a digital image of the lesion and processed using ImageJ [24]. For each *S. aureus* strain, at least 15 mice were assessed – the tissues for four of these mice were used for histological analysis and data for these mice were not available for CFU enumeration.

### Ethics Statement

All studies were reviewed and approved by the Animal Ethics Committee at the University of Melbourne approval number 0911248.2.

### Whole Genome sequencing of Australian CA-MRSA strains JKD6159 (ST93-IV [2B]), JKD6272 (ST1-IV [2B]), JKD6260 (ST1-IV [2B]) and JKD6177 (ST30-IV [2B])

We have previously described the sequencing, complete assembly and annotation of the genome of JKD6159, however the comparative genomics have not previously been reported [25]. Here, we also sequenced and partially assembled the genomes of three Australian CA-MRSA strains, using Illumina GAIIx 36 bp paired-end chemistry, yielding an average of 555 Mbp of reads per strain (∼200× coverage). These genomes were *de novo* assembled to draft form using Velvet 1.0 [26]. The reads from whole genome sequencing of these strains were also included in defining the core and accessory genome of JKD6159. The sequences are accessible from the NCBI Sequence Read Archive under accession SRA026511.1.

### Analysis of the JKD6159 genome sequence

A read mapping approach was developed to define a *S. aureus* core genome and the accessory genome for a given strain. Sequence reads from each *S. aureus* strain were aligned separately to the completed JKD6159 reference genome using SHRiMP 2.0 [27]. Publically available completed genomes (Table 2) were shredded into 75 bp reads at 25× coverage to produce a set of synthetic sequence reads. Those positions in JKD6159 that were covered by at least one read from each and every genome defined a *S. aureus* core genome. Reads from JKD6159 that did not map to any S. *aureus* genome (Table 2) were used to define JKD6159 regions of difference. Reads from any strain that did not map to the core genome represented that strain's accessory genome. SNPs were identified using Nesoni v0.35, which used the sequence reads for each genome aligned to the above-defined core genome to construct a tally of putative differences at each nucleotide position, including substitutions, insertions, and deletions (www.bioinformatics.net.au). This tally was then employed in a simple Bayesian model to decide whether a base (or deletion) could be called for the position, and if so, whether it differed from the reference sequence. A similar procedure was used to determine the presence or absence of insertions between positions in the reference. NCBI BLASTn+, Artemis and BRIG were also used for comparisons of JKD6159 with selected *S. aureus* genomes [28] (http://sourceforge.net/projects/brig/). SNPs were identified in the accessory genome elements pMW2, φSA2, and SCC*mec*IV for strains carrying them by read mapping using Nesoni v0.35 against reference sequences of each element taken from *S. aureus* MW2. Phylogenetic analyses were performed using a distance method, based on pairwise nucleotide sequence alignments for the *S. aureus* core genome among all strains or selected accessory elements among some strains. Split decomposition analysis was employed using uncorrected p distances with bootstrapping as implemented in SplitsTree4 [29].

**Table 2:** *Staphylococcus aureus* completed genomes used in this study.

	Genome	Multilocus Sequence Type (Clonal Complex)	Reference
1	NCTC8325	8 (8)	[48]
2	N315	5 (5)	[49]
3	Mu50	5 (5)	[49]
4	Mu3	5 (5)	[50]
5	MW2	1 (1)	[37]
6	USA300 FPR3757	8 (8)	[38]
7	USA300 TCH1516	8 (8)	[39]
8	MRSA252	36 (30)	[51]
9	MSSA476	1 (1)	[51]
10	RF122	151	[52]
11	Newman	8 (8)	[53]
12	COL	250 (8)	[54]
13	JH1	105 (5)	[55]
14	JH9	105 (5)	[55]
15	ED98	5 (5)	[56]
16	TW20	239 (8)	[57]
17	04-02981	225 (5)	[58]
18	S0385	398	[59]
19	JKD6008	239 (8)	[60]

### Statistical analysis

Kaplan Meier plots of *G. mellonella* killing results were analysed using the log rank test. Percentage mouse weight change at day 5, viable counts of *S. aureus* in mouse tissues and skin lesion area of each isolate versus JKD6159 were analyzed using the Mann Whitney test. All analyses were performed using Prism 5 for Macintosh v5.0b (GraphPad Software Inc.).

## Results

### Clinical Details

**Figure 1:**
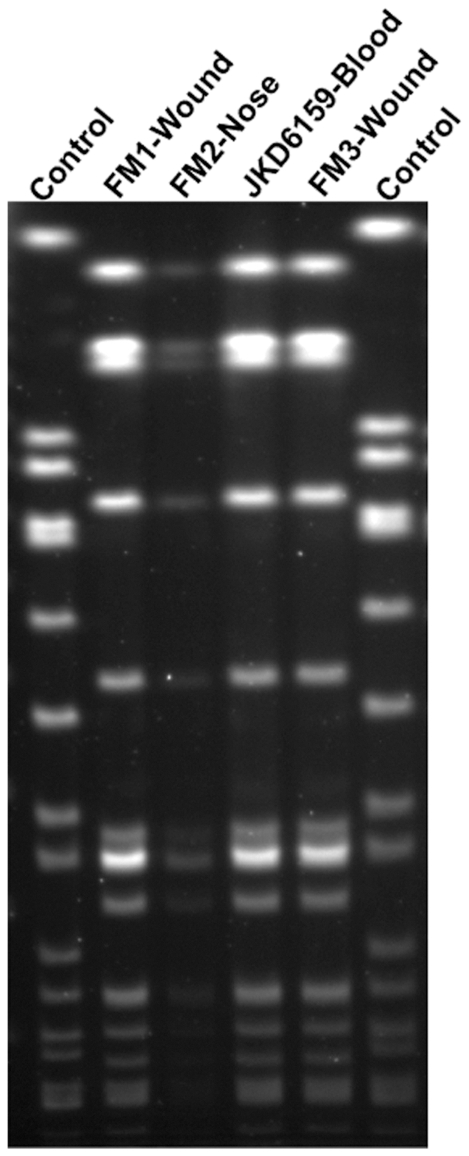
*Sma*I pulsed field gel electrophoresis analysis. PFGE of index isolate, *S. aureus* JKD6159 and isolates from three family members, FM1, FM2, FM3.

*Staphylococcus aureus* JKD6159 was isolated from the blood of a young male intravenous drug user with no prior health care exposure, who presented with a severe sepsis syndrome in 2004. He had cavitating pulmonary lesions, polyarticular septic arthritis and multiple deep-seated muscle abscesses. The isolate was resistant to methicillin, but susceptible to erythromycin, clindamycin, tetracycline, ciprofloxacin, rifampicin, fusidic acid and vancomycin. The patient required extensive surgical debridement and prolonged antimicrobial therapy with vancomycin and subsequently, linezolid, but eventually made a full recovery. In addition, the patient reported multiple household family members with recurrent skin infections. The *Sma*I PFGE pattern was identical between JKD6159 and the *S. aureus* isolates from three other family members, indicating an intra-familial outbreak with the same clone (Figure 1). Further typing demonstrated that JKD6159 was ST93-IV [2B]. This isolate was representative of the Australian CA-MRSA Queensland clone.

*S. aureus* strain JKD6272 was isolated from the blood of a woman with pneumonia and septic shock who had no prior hospitalizations. The isolate was resistant to methicillin and fusidic acid but susceptible to erythromycin, clindamycin, tetracycline, ciprofloxacin, rifampicin, trimethoprim and vancomycin. Further typing demonstrated that JKD6272 was ST1-IV [2B], and PVL negative. This isolate was representative of the Australian CA-MRSA clone WA-1.

*S. aureus* strain JKD6260 was isolated from a wound swab from a patient with a skin infection. The isolate was resistant to methicillin, fusidic acid and ciprofloxacin, but susceptible to erythromycin, clindamycin, tetracycline, rifampicin, mupirocin, trimethoprim and vancomycin. Typing demonstrated that JKD6272 was ST1-IV [2B], and PVL positive.

*S. aureus* strain JKD6177 was isolated from the blood of a patient with mitral valve endocarditis. He had recurrent skin infections with MRSA prior to presentation. The isolate was resistant to methicillin, but susceptible to erythromycin, clindamycin, tetracycline, ciprofloxacin, rifampicin, fusidic acid and vancomycin. Typing demonstrated that JKD6177 was ST30-IV [2B], and PVL positive. This isolate was representative of the SouthWest Pacific clone of CA-MRSA.

### JKD6159 exhibits high virulence in two animal models

**Figure 2:**
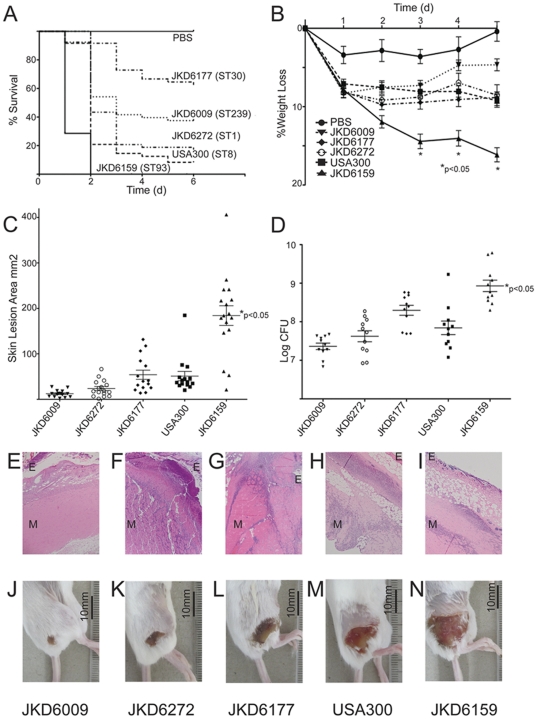
Virulence characteristics of *S. aureus* isolates. *S. aureus* JKD6159 (ST93-IV [2B], CA-MRSA) compared to four other MRSA strains. (A) *Galleria mellonella* virulence assay. Kaplan-Meier plot showing the percent survival of larvae injected with *S. aureus* strains JKD6159, JKD6009 (ST239-III [3A], hospital-associated MRSA), JKD6272 (ST1-IV [2B], CA-MRSA), JKD6177 (ST30-IV [2B], CA-MRSA), FPR3757 USA300 (ST8-IV [2B], CA-MRSA) up to 6 days post injection. The reduced survival time for larvae infected with JKD6159 compared to all other strains was significant (p<0.0001). (B) BALB/c mouse skin infection assay. Weight loss induced by intradermal infection with *S. aureus* strains is demonstrated as percentage loss of weight over 5 days. The difference in percentage weight loss between JKD6159 and all other strains was significant (p<0.05). At least 15 mice were used for each bacterial strain. Data shown are mean weight loss and SEM. (C) BALB/c mouse skin infection assay. Skin lesion area (mm^2^) at 5 days after infection was greatest with JKD6159 infected mice (p<0.05). Data shown are mean area and SEM. (D) BALB/c mouse skin infection assay. Recovery of *S. aureus* (log CFU) from infected tissues at 5 days after infection was greatest with JKD6159 infected mice (p<0.05). Data shown are mean CFU and SEM. (E-I) BALB/c mouse skin infection assay. Representative H&E stained histology sections from infected tissues at 100× magnification from JKD6009, JKD6272, JKD6177, USA300 and JKD6159 respectively. There was no significant difference in degree of inflammation observed in the CA-MRSA strain infections. However, mice infected with JKD6009, the hospital-associated MRSA strain had less marked inflammation and only mild myositis. (J-N) BALB/c mouse skin infection assay with representative macroscopic appearance of lesions resulting from infection with JKD6009, JKD6272, JKD6177, USA300 and JKD6159 respectively.

The reports of severe infections associated with ST93-IV [2B], and the severity of disease in our patient led us to compare the virulence of JKD6159 with other well-characterized clones of *S. aureus* (Table 1). All *S. aureus* strains tested had the same growth curve characteristics, indicating that any differences in virulence were not due to differential growth kinetics (Figure S1). However, JKD6159 was significantly more virulent than other community and healthcare-associated MRSA strains, including the USA300 strain FPR3757, in both an invertebrate and mouse infection model of *S. aureus* infection. In the *Galleria mellonella* larvae assay, JKD6159 caused significantly more rapid time-to-death than all other *S. aureus* strains (p<0.001, Fig. 2A). The differential virulence seen in the larvae assay was mirrored by the results of a mouse skin infection assay. Mice infected with JKD6159 developed large skin lesions and lost significantly more weight over a 5-day experimental period than mice infected with the other strains (p<0.05, Figure 2B-C). In line with these findings, the bacterial load in mouse tissues was highest in JKD6159-infected mice (p<0.05, Figure 2D). Histopathological analysis of mouse tissues showed that all CA-MRSA strains caused extensive epidermal and fat necrosis with myositis including in some cases myonecrosis, whilst infection with the hospital-associated MRSA strain JKD6009 caused less severe inflammatory changes with only mild myositis. Representative sections are shown in Figure 2E-I.

### Comparative genome analysis of JKD6159

**Figure 3:**
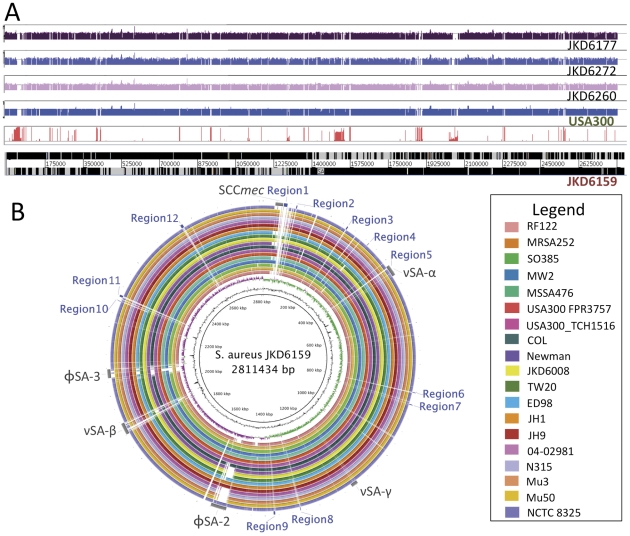
Whole genome sequence analysis and comparison of JKD6159 with other *S. aureus* strains. (A) Artemis linear view of JKD6159 chromosome, with vertical red bars identifying the position of accessory genome elements as determined by read mapping against 19 publicly available completed genomes and 62 unpublished, partially assembled genome sequences. Increasing height of vertical red lines indicates increasing specificity for JKD6159. Shown above the accessory genome analysis are the mapped positions of the short-reads obtained from Illumina sequencing CA-MRSA strains JKD6177, JKD6272 and JKD6260. Depicted also is an example of the synthetic reads obtained from completed whole genome sequences (USA300 shown here) to facilitate the comprehensive read mapping approach described in the methods. (B) Circular diagram of the JKD6159 chromosome showing (from inner to outer), % G+C, GC skew and the homology based on BLASTn+ analysis of JKD6159 to 19 completed *S. aureus* genomes (refer color-coded legend). Outer circle shows the location of accessory elements (grey) and the 12 regions of difference (blue) not present in the other *S. aureus* genomes examined.

**Figure 4:**
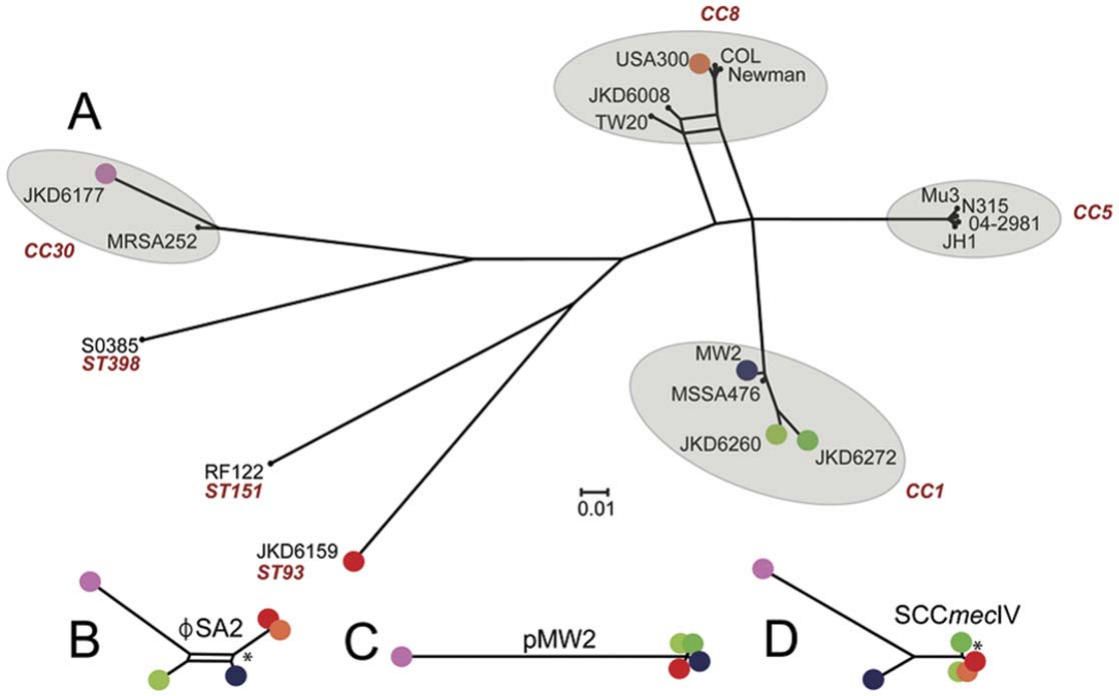
Phylogenetic analysis of *S. aureus* strains. Phylogenetic analysis based on the core genomes of *S. aureus* strains and selected accessory elements from CA-MRSA strains. (A) *S. aureus* phylogeny inferred by split decomposition analysis from pairwise comparisons of the 78,967 variable nucleotide positions identified from the core chromosome sequences of 20 completed *S. aureus* chromosomes and the 62 *S. aureus* strains for which unassembled reads were available, including the CA-MRSA strains sequenced in this study, JKD6177, JKD6260, JKD6272. CA-MRSA strains are indicated in colour (red JKD6159, pink JKD6177, orange FPR3757 USA300, blue MW2, light green JKD6260, dark green JKD6272) (B-D), Phylogenetic relationship of φSA2, pMW2 and SCC*mec*IV inferred by split decomposition analysis of nucleotide differences for each of these accessory elements between CA-MRSA strains. Scale bar indicates the number of nucleotide substitutions per site. All nodes have 100% bootstrap support except those indicated by asterisk (*) which have >60% support (1000 replicates). CC: clonal complex. ST: sequence type.

To gain insight into the high virulence of *S. aureus* ST93 strain JKD6159 we fully sequenced, assembled and annotated its genome and used it as a reference genome for comprehensive comparative genomics [25]. The JKD6159 genome consisted of a single 2,811,434 bp circular chromosome and a 20,730 bp circular, pMW2-like plasmid. For comparison, we also sequenced the genomes of three other Australian CA-MRSA isolates (JKD6272, JKD6260 & JKD6177) that were representative of the commonly occurring ST1 and ST30 clones (Table 1, Table S1, Figure 3A). Whole genome DNA∶DNA comparisons showed that the architecture of the JKD6159 chromosome was conserved compared to other *S. aureus* strains (Figure 3B). Using a read-mapping approach to objectively and comprehensively define the core and accessory genome of JKD6159, we found that 2,302,644 bp of JKD6159 (81.9%) was absolutely conserved amongst all 20 *S. aureus* strains with fully assembled genomes and 62 strains for which only unassembled sequence data was available (Table 2, Table S1, Figure 3). Pairwise comparisons of the concatenated 2,302,644 bp JKD6159 core genome with the core genomes of other *S. aureus* strains uncovered 78,967 variable nucleotide positions among all strains and permitted the construction of a high-resolution phylogeny by split decomposition analysis that confirmed the divergent genotype of ST93 (Figure 4A). ST93 was clearly divergent from other *S. aureus* strains with 7616 single nucleotide polymorphisms (SNPs) distinguishing this strain from other genomes. In comparison, the USA300 genome contained only 167 SNPs that varied from other strains. The JKD6159 core genome, like other *S. aureus* genomes, contained the genes encoding the important virulence factors Hla and α-type PSMs. The genes encoding gamma-hemolysin were also present.

Using the read mapping approach, we determined that 18.1% of the JKD6159 chromosome constituted its accessory genome (Figure 3A), a proportion that is consistent across all *S. aureus* genomes (range 13.2% [RF122]−18.1% [JH1]) and is also consistent with previous assessments based on microarray analyses [30], [31]. For a strain associated with severe clinical disease and high virulence in animal models, the accessory genome of JKD6159 was notable for the absence of common virulence-associated factors such as toxic shock syndrome toxin, exfoliative toxins and most of the staphylococcal enterotoxins; a finding consistent with an earlier report [32]. There were no recognized pathogenicity islands in JKD6159 although it contained the *S. aureus* genomic islands, νSAα, νSAβ and νSAγ (Figure 4B). Within νSAα there was a complement of tandem lipoprotein genes and eleven staphylococcal superantigen-like (*ssl*) genes, however *ssl9* appeared to be a pseudogene. Two of the 11 *ssl* CDS, *ssl3* (SAA6159_00377) and *ssl4* (SAA6159_00378), encoded proteins with predicted N-terminal sequences that demonstrated significant variation from orthologs in other strains. Although the exact role of SSLs is still being defined, they play a role in modulating the host immune system; in particular neutrophil and complement activation, both of which are key to human defense against *S. aureus* [33]. SSLs have been shown to be upregulated in some CA-MRSA strains [34]. We postulate that these variant SSLs may in part contribute to the strain's increased virulence.

In νSAβ, like other *S. aureus* genomes, JKD6159 had a cluster of serine protease genes and *lukDE*. However, unlike MW2 and FPR3757 USA300, the bacteriocin gene cluster was located elsewhere. In each of νSAα and νSAβ there were loci harboring distinctive type I restriction-modification systems. Restriction-modification systems attack foreign DNA, restricting DNA transfer [35], [36]. Type I systems consists of enzyme subunits which are encoded by the genes *hsdR, hsdM and hsdS.* The HsdS (specificity) subunit contains target recognition domains (TRD1 and TRD2) that allow for target sequence specificity. In JKD6159 the *hsdS* genes in each of these regions (SAA6159_00387 and SAA6159_01737) contained previously unreported TDR1 and TDR2 domains. Additionally, there was a third restriction modification system with a distinct *hsdS* gene (SAA6159_00055) in a novel 10 kb locus adjacent to SCC*mec* (Table S2). These differences in the restriction-modification system of JKD6159 may explain its relative resistance to the uptake of foreign DNA that we have observed during laboratory manipulation of this strain (unpublished observations).

JKD6159 contained two prophages φSA2 and φSA3. As in the other major CA-MRSA clones, φSA2 habours *lukSF-PV* [1], the genes that encode the two component pore-forming Panton-Valentine leukocidin (PVL). JKD6159 also contained φSA3, which is present in many other *S. aureus* genomes including MW2 (Figure 3B). This bacteriophage inserts into and disrupts the beta-hemolysin gene. In JKD6159, φSA3 contained genes encoding staphylokinase, and chemotaxis inhibitory protein but unlike MW2, did not harbor enterotoxin genes. A gene encoding an enterotoxin-like protein similar to that in RF122 (SAB0026) was also present in JKD6159.

The accessory genome of JKD6159 also contained 12 regions not present in the 19 other published, complete *S. aureus* genomes. These regions spanned 40,776 bp of the chromosome. The 32 CDS contained therein are listed in Table S2 and lie within segments identified as Regions 1–12 (Figure 3B, Table S2). JKD6159 also contained two copies of a Group II intron (Regions 6 & 11) that to our knowledge is the first example of this type of mobile DNA element in *S. aureus*.

As expected from its antibiotic phenotype, JKD6159 had few antibiotic resistance elements. It contained the SCC*mec*IV element found in other CA-MRSA strains but it did not have the adjacent arginine catabolic mobile element (ACME) seen in USA300. JKD6159 contained a *bla* operon located on a plasmid. This plasmid was identical to pMW2 and pSAS (from MSSA476). This plasmid was also highly similar to that carried on other community *S. aureus* strains including pWBG757 (NCBI Accession GQ900397.1), pSAP073A (NCBI Accession GQ900425.1), pWBG763 (NCBI Accession GQ900467.1), and pWBG750 (NCBI Accession GQ900392.1). Other mobile DNA present in JKD6159 included six intact copies and four partial copies of the insertion sequence element IS*Sau*3.

### Comparison of JKD6159 with other CA-MRSA

Comparisons of JKD6159 with the three other complete, publicly available genomes of CA-MRSA (MW2, USA300 FPR3757, USA300 TCH1516) [37], [38], [39], and the unassembled sequences for CA-MRSA strains JKD6272, JKD6260 & JKD6177, highlighted φSA2, pMW2 and SCC*mec*IV as elements common to these CA-MRSA strains, although JKD6272 did not contain φSA2, the phage bearing *lukSF-PV* required for expression of Panton Valentine Leukocidin (PVL). While high-resolution phylogenomic analysis based on SNP comparisons of the 2,302,644 bp *S. aureus* core genome demonstrated that some of these strains were distantly related to each other (Figure 4A), the same analysis based on SNP comparisons of only the accessory elements identified more limited sequence differences and produced individual tree topologies that suggested all strains except JKD6177 had recently acquired these elements (Figure 4B-C). Most striking was the limited sequence diversity among SCC*mec*, with 0.4 SNPs/100 bp for all CA-MRSA strains compared with 3.4 SNPs/100 bp across the entire chromosome for the same set of strains. These data are consistent with the contemporary acquisition of SSC*mec* by these diverse strains from a common source and the recent emergence of the global CA-MRSA phenomenon.

## Discussion

ST93 MRSA is now the most common CA-MRSA clone in Australia [14]. The reasons for its recent emergence are unknown but this increase is consistent with a worldwide spread of other global CA-MRSA clones. ST93 MRSA causes severe clinical disease that appears to mimic USA300. Little is known about ST93 although MLST and other typing methods have shown that ST93 is distinct to other *S. aureus* clones [16]. To understand the genetic basis for its recent emergence, we have compared both the virulence and genome of a recent clinical ST93 MRSA isolate (JKD6159) with other CA-MRSA including USA300.

We have demonstrated that ST93-IV [2B] was highly virulent in both the *Galleria* larvae virulence assay and mouse skin infection model. We used the *Galleria* model as a screening assay and validated our results in the mouse skin infection model. The *Galleria* killing assay has been previously used to assess the virulence of *S. aureus* [23], [40]. *Galleria mellonella* larvae are easily reared to large numbers, they can be maintained at 37°C and the assay gives consistently reproducible results. Furthermore, whilst the precise mechanisms by which *S. aureus* kills *Galleria* larvae remain uncertain, our *Galleria* assay time-to-death rates were comparable to the more conventional BALB/c mouse skin infection results. The skin infection assay was chosen because it approximates the most common disease presentation associated with the ST93 clone. The mouse skin infection model is a well-validated model and the histological lesions that result from staphylococcal infection are comparable to human staphylococcal skin infection [3], [4], [7], [8], [9]. The murine model for staphylococcal infections has some limitations in that mouse neutrophils are less susceptible to the effects of PVL than human and rabbit neutrophils [5], [10], [41]. Nonetheless, the pathology of murine skin infection caused by *S. aureus* is similar to that seen in rabbit staphylococcal skin infection [42].

While the whole genome sequence of JKD6159 demonstrated a relative paucity of recognizable virulence determinants, this ST93 strain did contain genes encoding the three most important CA-MRSA virulence factors, namely Hla, PVL and α-type PSMs [4], [5], [6], [7], [8], [10]. Given that we found few novel genes and no apparent candidates within these to explain the observed heightened virulence of JKD6159, it is possible that there are small genetic changes such as SNPs which lead to regulatory changes and therefore altered gene expression that are the reason for the observed increased virulence of JKD6159 compared to other CA-MRSA including USA300. These subtle mutations could impact known global regulators such as *agr*, or even other regulatory systems including sRNAs. The importance of SNPs that alter gene expression has been previously demonstrated in USA300 [12]. Investigations are underway to determine the relative contribution of SNPs to the enhanced virulence of JKD6159 using large-scale comparative genomics and virulence assessment approaches. Experiments are also in progress to determine the relative expression of Hla, PVL and α-type PSMs in ST93 compared to other CA-MRSA clones.

Because recent studies by others using *spa* gene high-resolution melt curve profiles have demonstrated genetic diversity within ST93 [43], we are currently examining additional methicillin-susceptible and methicillin-resistant ST93 isolates to determine whether the observations reported here in JKD6159 are representative of ST93 as a whole. In addition to assessing the generalizability of our findings, these studies will also be used to exploit any phenotypic differences we find through more extensive comparative and functional genomics to further enhance our understanding of the molecular basis for the emergence and enhanced virulence of ST93 CA-MRSA.

Our implementation of DNA sequence read mapping enabled us to accurately define at the nucleotide level, the core and accessory genome of *S. aureus* JKD6159. This approach is not biased by decisions based on orthology of protein coding sequences (CDS) and enables more precise quantification of differences and similarities between genomes. In this manner we demonstrated that the accessory genome constitutes between 13.2% [RF122] to 18.1% [JH1 and JKD6159] of the *S. aureus* chromosome. Read mapping also uncovered that JKD6159 shared elements with other CA-MRSA namely the PVL phage φSA2, pMW2 and SCC*mec*IV.

Comparative genomics supports the association previously reported between PVL and genetically disparate CA-MRSA *S. aureus* clones, and hence suggests a possible role for the PVL-containing phage in the emergence of community *S. aureus* infections. The PVL toxin has been associated epidemiologically with severe clinical presentations such as necrotizing pneumonia [44]. The laboratory evidence for its importance has focused on its role in staphylococcal virulence and the results of these studies are seemingly contradictory [3], [4], [5], [9], [45], [46]. We found that JKD6159 (PVL positive) showed the highest comparative virulence in both animal models (Figure 2). Interestingly, the PVL negative clone JKD6272 was as virulent as the other non-ST93 PVL strains including USA300, suggesting that the enhanced virulence of JKD6159 in our animal models may be PVL independent. The role of PVL and other genes encoded on φSA2 in dissemination and spread of *S. aureus* remains frustratingly uncertain.

Overall, the close relationship among distinct accessory genome elements including φSA2, pMW2 and SCC*mec*IV, in otherwise distantly related global *S. aureus* strains (which are separated by large distances in space and time) is an intriguing finding, and indicates the very recent acquisition of these elements from a common source by horizontal gene transfer. This association infers, but does not prove, a possible role for these elements in the fitness-for-spread and virulence of these strains in the community. These observations also suggest there are common strong selective pressures acting on genetically diverse *S. aureus* populations that are driving these specific gene acquisition events. These factors merit further investigation as they may hold clues for control of this global public health problem.

## Supporting Information

Figure S1 **Growth rates of** ***S. aureus*** **strains.** Comparative growth rates of *S. aureus* strains used in this study, showing no significant differences in growth characteristics between the strains.
(DOC)

Table S1 **Published, incomplete** ***S. aureus*** **genome sequences used in this study and mean read coverage against** ***S. aureus*** **JKD6159.**
(DOC)

Table S2 **Genomic Regions of Difference (1) - (12) in** ***S. aureus*** **strain ST93 JKD6159 compared to other non-ST93** ***S. aureus*** **strains.**
(DOC)
