# Supplementary figures and images for: The Dominant Australian Community-Acquired Methicillin-Resistant Staphylococcus aureus Clone ST93-IV [2B] Is Highly Virulent and Genetically Distinct
Source: PLoS One. 2011 Oct 3;6(10):e25887. doi: 10.1371/journal.pone.0025887 (PMC3185049; doi:10.1371/journal.pone.0025887)

**Supporting Information Figure S1:**


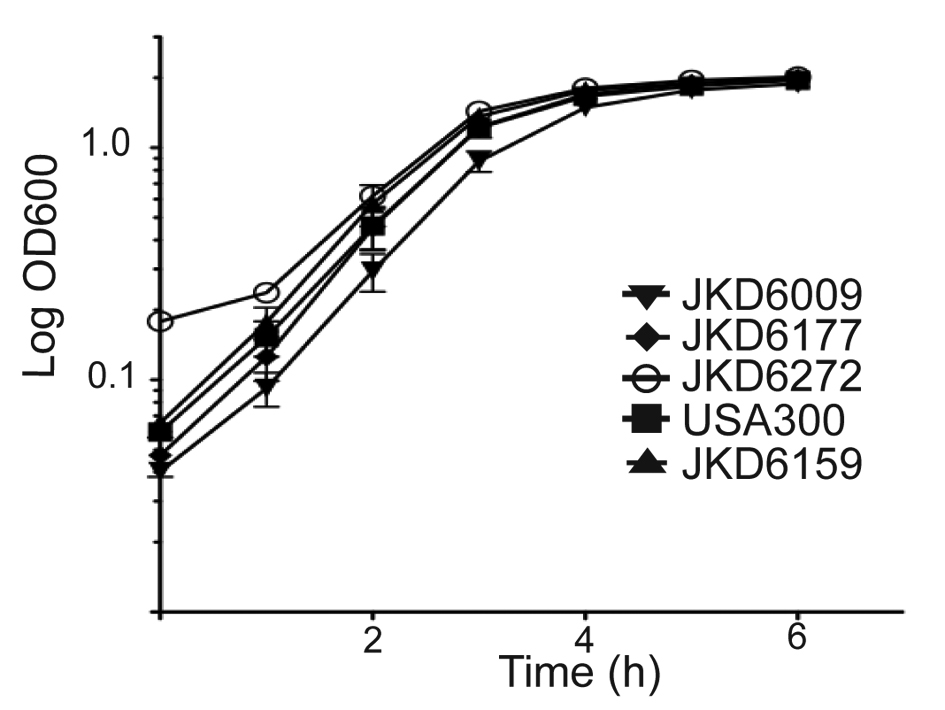

Supplement: Figure S1 — Growth rates of S. aureus strains. Comparative growth rates of S. aureus strains used in this study, showing no significant differences in growth characteristics between the strains. (DOC) [file pone.0025887.s001.doc]
