# Supplementary material for: The Dominant Australian Community-Acquired Methicillin-Resistant Staphylococcus aureus Clone ST93-IV [2B] Is Highly Virulent and Genetically Distinct
Source: PLoS One. 2011 Oct 3;6(10):e25887. doi: 10.1371/journal.pone.0025887 (PMC3185049; doi:10.1371/journal.pone.0025887)
Supplement: Table S1 — Published, incomplete S. aureus genome sequences used in this study and mean read coverage against S. aureus JKD6159. (DOC) [file pone.0025887.s002.doc]

**Supporting Information Table S1**

| **Strain Identification** | **Reference** | **Mean Coverage** |
| --- | --- | --- |
| MRSA_2A8 | [1] | 29.93 |
| MRSA_AGT1 | [1] | 28.95 |
| MRSA_AGT120 | [1] | 27.77 |
| MRSA_AGT67 | [1] | 32.66 |
| MRSA_AGT9 | [1] | 28.44 |
| MRSA_ANS46 | [1] | 23.26 |
| MRSA_BK2421 | [1] | 20.59 |
| MRSA_BRA2 | [1] | 30.16 |
| MRSA_BRA36 | [1] | 23.41 |
| MRSA_BZ48 | [1] | 26.09 |
| MRSA_CHI59 | [1] | 24.29 |
| MRSA_CHI61 | [1] | 23.35 |
| MRSA_CHL151 | [1] | 26.49 |
| MRSA_DEN907 | [1] | 27.39 |
| MRSA_FFP103 | [1] | 19.5 |
| MRSA_GRE108 | [1] | 15.8 |
| MRSA_GRE18 | [1] | 11.29 |
| MRSA_GRE317 | [1] | 15.28 |
| MRSA_GRE4 | [1] | 17.87 |
| MRSA_HGSA142 | [1] | 29.5 |
| MRSA_HGSA9 | [1] | 12.85 |
| MRSA_HSA10 | [1] | 22.75 |
| MRSA_HSJ216 | [1] | 23.52 |
| MRSA_HU106 | [1] | 14.12 |
| MRSA_HU109 | [1] | 19.11 |
| MRSA_HU25 | [1] | 21.1 |
| MRSA_HUR18 | [1] | 11.92 |
| MRSA_HUSA304 | [1] | 14.1 |
| MRSA_ICP5011 | [1] | 35.86 |
| MRSA_ICP5014 | [1] | 39.23 |
| MRSA_ICP5062 | [1] | 16.13 |
| MRSA_LHH1 | [1] | 28.84 |
| MRSA_S102 | [1] | 24.85 |
| MRSA_S106 | [1] | 31.28 |
| MRSA_S130 | [1] | 16.81 |
| MRSA_S2 | [1] | 26.23 |
| MRSA_S21 | [1] | 20.96 |
| MRSA_S24 | [1] | 21.8 |
| MRSA_S25 | [1] | 34.37 |
| MRSA_S26 | [1] | 42.05 |
| MRSA_S38 | [1] | 24.97 |
| MRSA_S39 | [1] | 28.3 |
| MRSA_S40 | [1] | 29.71 |
| MRSA_S42 | [1] | 16.42 |
| MRSA_S7 | [1] | 49.09 |
| MRSA_S71 | [1] | 34.56 |
| MRSA_S78 | [1] | 28.29 |
| MRSA_S81 | [1] | 28.25 |
| MRSA_S85 | [1] | 16.03 |
| MRSA_S87 | [1] | 24.86 |
| MRSA_S93 | [1] | 24.33 |
| MRSA_TUR1 | [1] | 23.56 |
| MRSA_TUR27 | [1] | 20.64 |
| MRSA_TUR9 | [1] | 14.97 |
| MRSA_URU110 | [1] | 31.7 |
| MRSA_URU34 | [1] | 25.02 |
| Sa_JKD6177 | This study | 100.4 |
| Sa_JKD6210 | [2] | 228.61 |
| Sa_JKD6229 | [2] | 207.06 |
| Sa_JKD6009 | [3] | 22.70 |
| Sa_JKD6260 | This study | 69.04 |
| Sa_JKD6272 | This study | 186.71 |

References:

1. Harris SR, Feil EJ, Holden MT, Quail MA, Nickerson EK, et al. (2010) Evolution of MRSA during hospital transmission and intercontinental spread. Science 327: 469-474.

2. Gao W, Chua K, Davies JK, Newton HJ, Seemann T, et al. (2010) Two novel point mutations in clinical *Staphylococcus aureus* reduce linezolid susceptibility and switch on the stringent response to promote persistent infection. PLoS Pathog 6: e1000944.

3. Howden BP, Stinear TP, Allen DL, Johnson PD, Ward PB, et al. (2008) Genomic analysis reveals a point mutation in the two-component sensor gene *graS* that leads to intermediate vancomycin resistance in clinical *Staphylococcus aureus*. Antimicrob Agents Chemother 52: 3755-3762.
