# Supplementary material for: The Dominant Australian Community-Acquired Methicillin-Resistant Staphylococcus aureus Clone ST93-IV [2B] Is Highly Virulent and Genetically Distinct
Source: PLoS One. 2011 Oct 3;6(10):e25887. doi: 10.1371/journal.pone.0025887 (PMC3185049; doi:10.1371/journal.pone.0025887)
Supplement: Table S2 — Genomic Regions of Difference (1) - (12) in S. aureus strain ST93 JKD6159 compared to other non-ST93 S. aureus strains. (DOC) [file pone.0025887.s003.doc]

**Supporting Information Table S2**

| **Region,**  **Region Size**  **(Region Coordinates)** | **Locus Tag** | **Coordinates** | **Predicted Protein size (aa)** | **Predicted Product** | **Closest Ortholog** | **aa identity in overlap (%), size of overlap (aa)** | **Comment** |
| --- | --- | --- | --- | --- | --- | --- | --- |
| **Region 1**  10,611 bp  (60260-70871) | SAA6159_00052 | 615336–60619 | 305 | Conserved hypothetical protein | Hypothetical conserved protein *Mesorhizobium loti* | 27, 289 | This region is adjacent to SCC*mec*IV cassette and contains a restriction modification system |
|  | SAA6159_00053 | 62978-61530 | 483 | ATP-dependent endonuclease | Hypothetical conserved protein *Mesorhizobium loti* | 36, 472 |  |
|  | SAA6159_00054 | 63166-64680 | 505 | Type I site-specific deoxyribonuclease methyltransferase subunit, HsdM_3 | Site-specific DNA-methyltransferase *Staphylococcus epidermidis* BCM-HMP0060 | 89, 504 |  |
|  | SAA6159_00055 | 64670-65902 | 411 | Type I restriction modification DNA specificity protein, HsdS_3 | Hypothetical protein CLOSS21_01605 *Clostridium* sp. SS2/1 | 37, 410 |  |
|  | SAA6159_00056 | 65886-69014 | 1043 | Type I site-specific deoxyribonuclease restriction subunit | Type I restriction-modification system endonuclease homologue *Staphylococcus aureus* | 82, 1040 |  |
|  | SAA6159_00057 | 69127-69834 | 236 | Hypothetical membrane protein | Putative membrane protein *Staphylococcus warneri* L37603 | 30, 137 |  |
|  | SAA6159_00058 | 69827-70330 | 168 | Hypothetical protein | Hypothetical protein ThewiDRAFT_2042 *Thermoanaerobacter wiegelii* Rt8.B1 | 41, 62 |  |
|  | SAA6159_00059 | 70741-70562 | 60 | Hypothetical protein | Hypothetical protein SLGD_00417 *Staphylococcus lugdunensis* HKU09-01 | 50, 57 |  |
| **Region 2**  2385 bp  (96703-99087) | SAA6159_00088 | 97042-98505 | 488 | Hypothetical protein | NAD-dependent epimerase/dehydratase *Staphylococcus hominis* SK119 | 60, 504 |  |
|  | SAA6159_00089 | 98568-99062 | 165 | Hypothetical protein | Hypothetical protein SLGD_00183 *Staphylococcus lugdunensis* HKU09-01 | 59, 161 |  |
| **Region 3**  2768 bp  (254453-257220) | SAA6159_00211 | 255111-254560 | 184 | Transcriptional regulator | Predicted protein *Staphylococcus aureus* A9635 | 96, 183 | A9635 is a partially assembled *S. aureus* genome (NCBI Accession NZ_ACKI00000000) |
|  | SAA6159_00212 | 255265-255909 | 215 | NADH-dependent epimerase | NAD-dependent epimerase/dehydratase *Staphylococcus aureus* A9635 | 97, 214 |  |
|  | SAA6159_00213 | 255922-256824 | 301 | Hydrolase, alpha/beta superfamily | Dienelactone hydrolase *Staphylococcus aureus* A9635 | 99, 300 |  |
| **Region 4**  737 bp  (332323-333059) | SAA6159_00282 | 332478-333002 | 175 | Metal-dependent hydrolase | Metal-dependent hydrolase homologue *Staphylococcus fleurettii* | 55, 170 |  |
| **Region 5**  2190 bp  (422011-424201) | SAA6159_00372 | 423756-422302 | 485 | Transcriptional regulator | ATPase, AAA_4 family *Geobacter sulfurreducens* KN400 | 52, 481 |  |
| **Region 6**  1286 bp  (802238-803524) | SAA6159_00715 | 802238-803524 | 429 | Reverse transcriptase, group II intron | Regulatory protein GntR *Staphylococcus aureus* subsp. a*ureus* D139 | 99, 428 | Group II intron  D139 is a partially assembled *S. aureus* genome (NCBI Accession NZ_ACSR00000000 |
| **Region 7**  1514 bp  (838007-839521) | SAA6159_00744 | 838007-839521 | 505 | Staphylocoagulase precursor | von Willebrand factor-binding protein *Staphylococcus aureus*  A9635 | 76, 513 | A9635 is a partially assembled *S. aureus* genome (NCBI Accession NZ_ACKI00000000) |
| **Region 8**  1071 bp  (1296295-  1297366) | SAA6159_01171 | 1296926-1297168 | 81 | Conserved hypothetical protein | Hypothetical protein SATG_02718 *Staphylococcus aureus* subsp. *aureus* D139 | 79, 80 | D139 is a partially assembled *S. aureus* genome (NCBI Accession NZ_ACSR00000000) |
|  | SAA6159_01172 | 1297168-1297365 | 66 | Conserved hypothetical protein | Hypothetical protein SATG_02719 *Staphylococcus aureus* subsp. *aureus* D139 | 94, 65 |  |
| **Region 9**  4173 bp  (1371025-  1375198) | SAA6159_01246 | 1371846-  1371145 | 234 | ABC-type dipeptide/oligopeptide/  nickel transporter, ATPase component 2 | Oligopeptide permease *Staphylococcus aureus* A9635 | 96, 233 | A9635 is a partially assembled *S. aureus* genome (NCBI Accession NZ_ACKI00000000) |
|  | SAA6159_01247 | 1372612-1371839 | 258 | ABC-type dipeptide/oligopeptide/  nickel transporter, ATPase component 2 | Peptide ABC transporter *Staphylococcus aureus* A9635 | 97, 257 |  |
|  | SAA6159_01248 | 1373429-  1372599 | 277 | Oligopeptide ABC superfamily ATP binding cassette transporter, membrane protein | Oligopeptide transporter permease *Staphylococcus aureus* A9635 | 99, 276 |  |
| **Region 10**  2201 bp  (2277949-  2280150) | SAA6159_02109 | 2278694-  2277999 | 232 | Subtilase family protease | Peptidase S8 and S53 subtilisin kexin sedolisin *Bacillus thuringiensis* IBL 200 | 34, 210 |  |
|  | SAA6159_02111 | 2280052-2279303 | 250 | Hypothetical protein | Hypothetical protein SPSINT_1693 *Staphylococcus pseudintermedius*  HKU10-03 | 67, 193 |  |
| **Region 11**  4923 bp  (2290825-2295748) | SAA6159_02120 | 2292111-  2290825 | 429 | Reverse transcriptase, group II intron | Regulatory protein GntR *Staphylococcus aureus* subsp. *aureus* D139 | 99, 428 | D139 is a partially assembled *S. aureus* genome (NCBI Accession NZ_ACSR00000000) |
|  | SAA6159_02121 | 2292381-  2292172 | 70 | Hypothetical protein | No hits |  |  |
|  | SAA6159_02122 | 2295202-  2292908 | 765 | Conserved hypothetical protein | Hypothetical protein LMOf2365_0687 *Listeria monocytogenes* str. 4b F2365 | 36, 791 |  |
|  | SAA6159_02123 | 2295581-  2295748 | 56 | Hypothetical protein | Hypothetical protein Bcoam_15246 *Bacillus coahuilensis* m4-4 | 50, 28 |  |
| **Region 12**  6917 bp  (2561578-  2568495) | SAA6159_02388 | 2563191-  2561578 | 538 | Conserved hypothetical protein | Hypothetical protein STAWA0001_1449 *Staphylococcus warneri* L37603 | 73, 530 |  |
|  | SAA6159_02389 | 2565953-  2563287 | 889 | Cell-wall associated Sdr-like protein | Conserved hypothetical protein *Staphylococcus aureus* subsp. *aureus* USA300_TCH959 | 98, 313 | USA300_TCH959 is a partially assembled *S. aureus* genome (NCBI Accession NZ_AASB02000020.1) |
|  | SAA6159_02391 | 2566637-  2566128 | 170 | Hypothetical protein | Conserved hypothetical protein *Staphylococcus aureus* subsp. *aureus* USA300_TCH959 | 98, 71 |  |
|  | SAA6159_02392 | 2567263-  2566913 | 117 | Staphylococcal accessory regulator T, SarT_1 | Accessory regulator A family protein *Staphylococcus aureus* subsp. *aureus* USA300_TCH959 | 100, 115 |  |
